# Extracts from the Diary of an Old Physician

**Published:** 1837

**Authors:** 


### Art. III.—Extracts from the Diary of an old Physician.

*Various items and cases*, No. 2, continued from the 38th Number of this Journal.

### Indian Doctoring in Dysentery and Consumption.

In June 1835, attended on a female aged 48 years, full leuco-phlegmatic habit, with inflammation and sphacelus of the leg, between the gostrocnemii muscles and tendo achilles, consequent on an attack of synochial fever. Depletion was freely used, and cooling lotions, until the heat subsided. The sphacelated parts were removed, and further progress stayed by the application of lint, wet with spirits, tereb. and ol. olivarum, over which was laid a warm poultice of yeast, filled with pulverised charcoal. This application I have often found serviceable—considerable febrile excitement attended the course of the disease in the leg. While this woman was recovering, a daughter of her husband by a former wife, aged 18 years was attacked with dysentery, a strolling vagabond, too lazy to work, but calling himself "an Indian doctor," stopped at the house, and seeing the young woman in bed enquired her complaint. On hearing it was the dysentery, he told her father he could soon cure the disease with a few simples from the woods, boiled up and mixed with whiskey—as a large portion of mankind are fond of mystery, and have great faith in the medical knowledge of Indians, he was simple enough to employ him—accordingly a quart bottle was filled with a decoction of astringent and powerfully cathartic roots, made strong with whiskey, and the poor girl drenched for two or three days, until her life was despaired of—when they sent for a physician, but the disease had made too great progress to be arrested, and she died in a few hours, apparently a victim to ignorance and credulity.

The most singular case of deception and of the superstitious propensities of mankind, even in this enlightened land, took place in Essex county, Massachusetts, about twenty-five years ago. I was then studying medicine; a patient of my worthy teacher was lying in the last stage of a consumption. She was a young woman, and of a family predisposed to this disease, of which several had already died—at this juncture a strolling Indian doctor came into the neighborhood, and hearing of her sickness called at the house. With great solemnity he told her father that the course of her disease, and the continual wasting of her flesh was occasioned by a brother who had last died of the disease, three years before, and whose heart he affirmed was still fresh and plump, and drew in some mysterious manner its support from her blood. The remedy proposed, he said was infallible; which was to take up the dead body of her brother, separate the heart from its attachments, burn it to ashes; and let the sick woman drink it in a decoction which he would prepare from "roots and herbs." On the strength of this declaration and the most implicit faith in its fulfilment, the body was exhumed and search made for the sound and bleeding heart; but in vain, it had become a mass of corruption with the rest of the carcase, and the poor girl saved from the trial of such a disgusting remedy. Led by curiosity, I was present with several others, in the burying ground at the time, and recollect the look of disappointment exhibited by the father, when no heart could be found. With such examples of credulity in our own land, we need not wonder at the disgusting and unnatural remedies of the days of ignorance and superstition.

During the month of June several cases of dysentery came under my care, which were successfully treated by a free course of calomel purges, with sudorific powders of ipe cac.

opium and camphor; to which were added demulcent drinks and food—of the latter article however, the less the patient can possibly subsist on the better—many cases of this disease, are prolonged, and terminate fatally at last from too much nourishment. The bowels require repose, which can only be obtained by withholding from them their accustomed employment, the elimination of chyle from food. It is of more importance in this disease than many physicians are aware of. In July a number of cases of the common cholera appeared. In this disease after evacuating the stomach with warm gruel, or an emetic of epecacuanha if necessary, I have found the effervescing soda powders, with a little brandy and sugar, very excellent in allaying the irritable state of the stomach. Bottled cider, porter, or good beer will sometimes act like a charm in restoring the exhausted and prostrated powers of the system. If the extremities are cold, as they almost always are, the application of hot spirits with cayenne pepper, is a very useful article in recalling the blood to the cuticular vessels. If from bilious derangement, it must be treated with calomel in large doses, and the stomach restored to its usual healthy action with colombo and spices, in a simple watery infusion.

### Case of Hallucination.

In the fore part of July, attended on M. G. Aiken, a young man about 26 years of age, sanguineous habit, and cheerful disposition. He was a proficient on the violin and flute, and often practiced his art at dancing parties. He was now suffering with an attack of pleuritic fever, consequent on great bodily fatigue and want of sleep, having followed his vocation for two or three successive nights without any rest. The pain in his side was very acute and fever high, which were readily subdued by free venesection and cathartics. The vigilance however still continued, and nothing which I could give or devise, produced any sleep, although he had now been wide awake for four days and nights. He was continually in a pleasant delirium talking and laughing, and night and day busily occupied in some imaginary employment. His most favorite business was that of hunting wild bees, on which occasions he would jump out of bed, and act the bee hunter by drawing lines through the air across the room, to trace their flight. This hallucination continued for nearly a week, although the largest doses of opium, a pillow of hops, and various other sleep producing remedies were tried—at that time the various sulphates and acetates of morphia, were unknown in Ohio practice, or it is probable some one of them would have quieted the nervous excitement. He at length fell into a *natural sleep*, which continued for twenty-four hours from which he awoke in perfect health, excepting a general debility, which left him in a day or two—such cases are sometimes relieved by cold dashes to the head, while the feet are placed in hot water. It was tried here without benefit. It nearly resembled "mania a potu;" but in this instance the patient was not a regular toper, and now had drank but very little. It was an instance of pure phrenological excitement, continued after all febrile disease had ceased.

In August, cases of dysentery still continued in some instances combined with fever of a typhus type. In such attacks fine charcoal, combined with fresh lively yeast, was found to be eminently useful—opium, in free doses was very requsite. But few agues followed the fevers of this year, the systems of the sick seeming to be purging or freeing themselves from the miasm, so fertile in remittents during the years 1822 and 1823; which seasons will long be remembered in the valley of the Ohio.

### Painter's Colic.

In October saw a case of obstinate colic in J. K. R., a painter by trade, and a great drinker of whiskey. He had been sick three or four days, under the care of Dr. G., who had given repeated cathartics without any effect—on the 26th of the month I gave him four grains of pure opium at a dose, to quiet pain. Left him a box of Hull's famous colic pills, which are composed of various spices, calomel and active purgative gum-resins—with directions to take two [every hour until they operated, and to aid the movement by occasional enemata of warm gruel. 27th, Had taken all the pills, no operations, injections return as given without any fecal smell. Prepared eighty grains of calomel in three boluses, one to be taken every three hours. In addition to which, mixed up a tea-cup full of fine charcoal in treacle, the whole of which to be taken at once. His pulse being quite full, bled him very freely. 28th, Called, and found that he had retained all the medicine without any difficulty, although few stomachs are thus accommodating; and what was still better it had operated very freely, affording much ease, and relief of mind, as well as body--after six days and nights of continued suffering, no one can imagine the pleasurable sensations that follow a free catharsis, but him who has experienced it—as he still felt some uneasiness in the bowels, he was directed to take opium and asafœtida pills, sufficient to quiet them; and drink a decoction of sena, manna, and cr. tartar, under this course he soon recovered. In obstinate cases of cholic, with constipation, I have several times known finely pulverised charcoal to move the bowels when every other known remedy had failed. Latterly in these difficult cases, attended with vomiting, I have several times directed the use of *small lumps of ice*, slowly melted in the mouth, and swallowed with the happiest effect—allaying and quieting the irritable state of the stomach, and promoting the peristaltic motion without violence; so as greatly to assist the operation of the cathartic medicines. In two or three instances, I am fully persuaded that the use of this remedy, (which at first view all the old nurses and many doctors, would pronounce a very hazardous, if not a fatal medicine,) has been the means of saving the life of the patient.

*January* 6, 1837.

